# Soft tissue changes in upper incisors from tooth movements using a new measurement method based on digital scans superimposition in adult patients

**DOI:** 10.1007/s00784-026-06873-0

**Published:** 2026-04-23

**Authors:** Luis Sastre-Buades, Verónica García-Sanz, Beatriz Tarazona-Álvarez, Natalia Zamora-Martínez, Sara Camañes-Gonzalvo, José María Montiel-Company, Vanessa Paredes-Gallardo, Carlos Bellot-Arcís

**Affiliations:** 1 Department of Stomatology, University of Valencia, Valencia, Spain; 2 Department of Stomatology, Faculty of Medicine and Dentistry, University of Valencia, Valencia, Spain; 3 Department of Stomatology, Orthodontics Teaching Unit, Clínica Odontológica, C/ Gascó Oliag nº 1, Valencia, CP: 46010 Spain

**Keywords:** Superimposition, Alveolar bone, Gingival recession, Tooth movement

## Abstract

**Objective:**

To develop a novel digital method to quantify changes in the free gingiva following tooth movement, to measure upper incisor movements by superimposing pre- and post-treatment intraoral scans, and to explore the relationship between tooth movements and gingival changes.

**Materials and methods:**

An observational and descriptive-analytical study was conducted on 31 patients, analyzing 124 upper central and lateral incisors. Dental movements (protrusion, retrusion, retroclination, proclination, extrusion, intrusion, rotation, and inclination) and gingival changes (apical or coronal migration) were quantified by superimposing intraoral scans using GOM Inspect and Geomagic Wrap software.

**Results:**

Changes in crown proclination were associated with coronal displacement of the clinically visible gingival margin (*r* = 0.560), whereas changes in crown retroclination were associated with apical displacement (*r* = − 0.275). A positive association was also observed between apical displacement and age (*r* = 0.216). No significant associations were found for tipping, rotation, extrusion, or intrusion.

**Conclusions:**

A digital STL-based method was used to quantify crown tooth movement and relative changes in the position of the clinically visible gingival margin. Changes in crown proclination and retroclination were associated with coronal and apical displacement of the gingival margin, respectively, and should be interpreted as correlational findings.

**Clinical relevance:**

This digital method allows precise assessment of gingival changes during orthodontic treatment, helping clinicians predict and minimize soft tissue alterations related to incisor movements.

**Supplementary Information:**

The online version contains supplementary material available at 10.1007/s00784-026-06873-0.

## Introduction

The primary objective of orthodontic treatment is to correct malocclusions for both aesthetic and functional purposes; however, it carries risks associated with the remodeling of both hard and soft tissues. One significant side effect is alveolar bone dehiscence, a condition in which the dental root becomes exposed due to inadequate alveolar bone coverage, typically defined as a loss exceeding 2 mm from the cementoenamel junction. Alveolar dehiscence constitutes a predisposing factor for gingival recession, which is characterized by the apical displacement of the gingival margin. This condition frequently involves the anterior teeth and is commonly associated with a reduction in the width of keratinized gingiva [1–3].

Thus, it is essential to consider the anatomical limitations of the alveolar bone when planning orthodontic tooth movements in order to prevent adverse outcomes such as alveolar dehiscence, fenestration, or external root resorption. Theories describing bone remodeling during orthodontic treatment include the “tooth movement within the bone” concept, in which the tooth remains encased by alveolar bone, and the “tooth movement through the bone” model, where the tooth is displaced beyond the osseous housing, thereby increasing the risk of gingival recession.

Additional predisposing factors for recession include a thin periodontal biotype, absence of attached gingiva, and a reduced height of the alveolar crest due to dental misalignment [4]. Nevertheless, orthodontic treatment can serve as an adjunct to periodontal therapy, with the potential to enhance both the outcomes and the effectiveness of periodontal surgical procedures [5].

In recent years, several techniques have been employed to assess soft tissue alterations following periodontal or orthodontic interventions. These include non-invasive methods such as visual inspection and probe transparency assessment, as well as invasive approaches including transgingival probing and the use of periodontal calipers [6]. Although cephalometric radiographic superimposition has been widely utilized to evaluate orthodontic tooth movement, its application in assessing periodontal changes is limited [7]. Furthermore, cephalometric radiographs represent two-dimensional projections of three-dimensional anatomical structures, which are limited by factors such as image distortion, superimposition of anatomical landmarks, and unavoidable ionizing radiation exposure [7].

Digital technology has significantly transformed contemporary orthodontic practice, with intraoral scanners increasingly replacing conventional plaster models to generate high-precision digital casts [8]. These digital models offer advantages, including improved data storage, reduced risk of physical damage, and improved interdisciplinary communication among healthcare professionals [5]. Moreover, three-dimensional diagnostic software and stereolithography (STL) files enable detailed analysis of treatment outcomes through digital model superimposition. Nevertheless, accurate alignment of digital models remains a challenge, particularly in growing patients, due to ongoing palatal and mandibular development [9].

Although the effects of orthodontic movements on hard tissue are well-documented, their impact on soft tissues, particularly gingiva, remains unclear. The relationship between orthodontic movements and gingival changes, including recessions, requires further investigation [10]. Accurate model alignment and reference point selection are crucial for reliable assessments of both periodontal and orthodontic changes [5, 11].

This study aimed to develop a digital method using STL model superimposition to evaluate the impact of orthodontic tooth movements on gingival tissue. By quantifying gingival changes before and after treatment, it assessed the effects of different tooth movements on periodontal health. The research also sought to integrate orthodontic and periodontal approaches to prevent gingival recession and improve treatment outcomes.

Thus, the primary objective was to investigate the relationship between free gingiva changes and orthodontic tooth movements by comparing pre- and post-treatment STL models. Additionally, the study aimed to develop a novel digital method to quantify these gingival changes and to assess tooth movements in the upper incisors.

## Subjects and methods

### Overview

This retrospective observational and descriptive-analytical study was conducted on adult patients diagnosed with various malocclusions to evaluate changes in the marginal gingiva. The study was approved by the Research Ethics Committee of the University of Valencia and conducted in accordance with the Declaration of Helsinki (verification code W7AAW3QHXN73HE76). Reporting followed STROBE guidelines for observational research [12].

### Participants

All participants were treated at the University of Valencia (Valencia, Spain). Informed consent was obtained from all patients.

The inclusion criteria comprised adults aged 25 years or older, individuals with periodontal stability throughout orthodontic treatment, patients presenting with any type of malocclusion, availability of both pre-treatment and post-treatment STL records, and the presence of all four upper incisors. All patients were treated with clear aligners, and no fixed orthodontic appliances were used. Periodontal stability was maintained throughout treatment, with no clinical signs of gingival inflammation or edema at the time of intraoral scanning. In addition, only patients presenting mild to moderate maxillary anterior crowding were included. The exclusion criteria encompassed patients who had undergone interproximal reduction on any of the four upper incisors, those who had undergone gingivectomy, cases with severe crowding were excluded in order to avoid extensive dentoalveolar compensations that could influence periodontal outcomes, and individuals with congenital absence of anterior maxillary teeth.

### Orthodontic treatment

Three-dimensional STL models were obtained using the iTero Element 2^®^ intraoral scanner (Align Tech., Inc.) before and after treatment with clear aligners. All intraoral scans were acquired by the same experienced operator using a standardized scanning protocol to reduce operator-related variability. Standardized protocols addressed horizontal, vertical, and rotational discrepancies. Attachments were bonded using Transbond XT resin (3 M Unitek, CA, USA). Patients received oral hygiene instructions, and plaque scores were recorded using the Spanish Society of Periodontology and Osseointegration (SEPA) periodontogram. Regular follow-ups ensured adherence to the treatment plan. All patients demonstrated periodontal health throughout treatment and were regularly monitored using plaque indices to ensure adequate oral hygiene and absence of active gingival inflammation.

### Superimposition

The “University of Valencia method” for superimposition and quantification of dental movements and gingival changes is described in this study. Data from three-dimensional intraoral scans were exported in STL format and imported into Geomagic Wrap software (3D Systems, South Carolina, United States). The pre-treatment scan (T0) and the post-treatment scan (T1) with clear aligners were obtained.

**Fig. 1:**
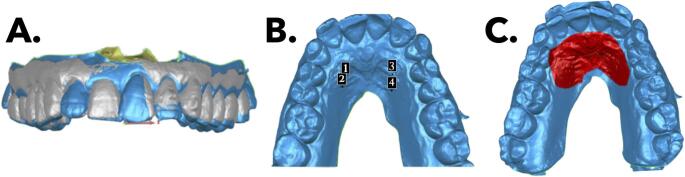
**A** Automatic superimposition; **B** Superimposition by points; **C** Superimposition by areas

Three types of superimpositions were performed: automatic, by points on the third palatine ridge, and by palatal areas (Fig. 1). The resulting 3D models were exported as STL files and analyzed in GOM Inspect software (GOM GmbH, Braunschweig, Germany) to measure dental and gingival changes in the upper incisors.

All reference planes and tooth axes were established on the pre-treatment model (T0) and subsequently applied to the post-treatment model (T1) following superimposition. Tooth movement and gingival margin measurements were performed along the same predefined sagittal section, with identical spatial orientation and without subjective point selection.

### Measurement of dental movements

**Fig. 2:**
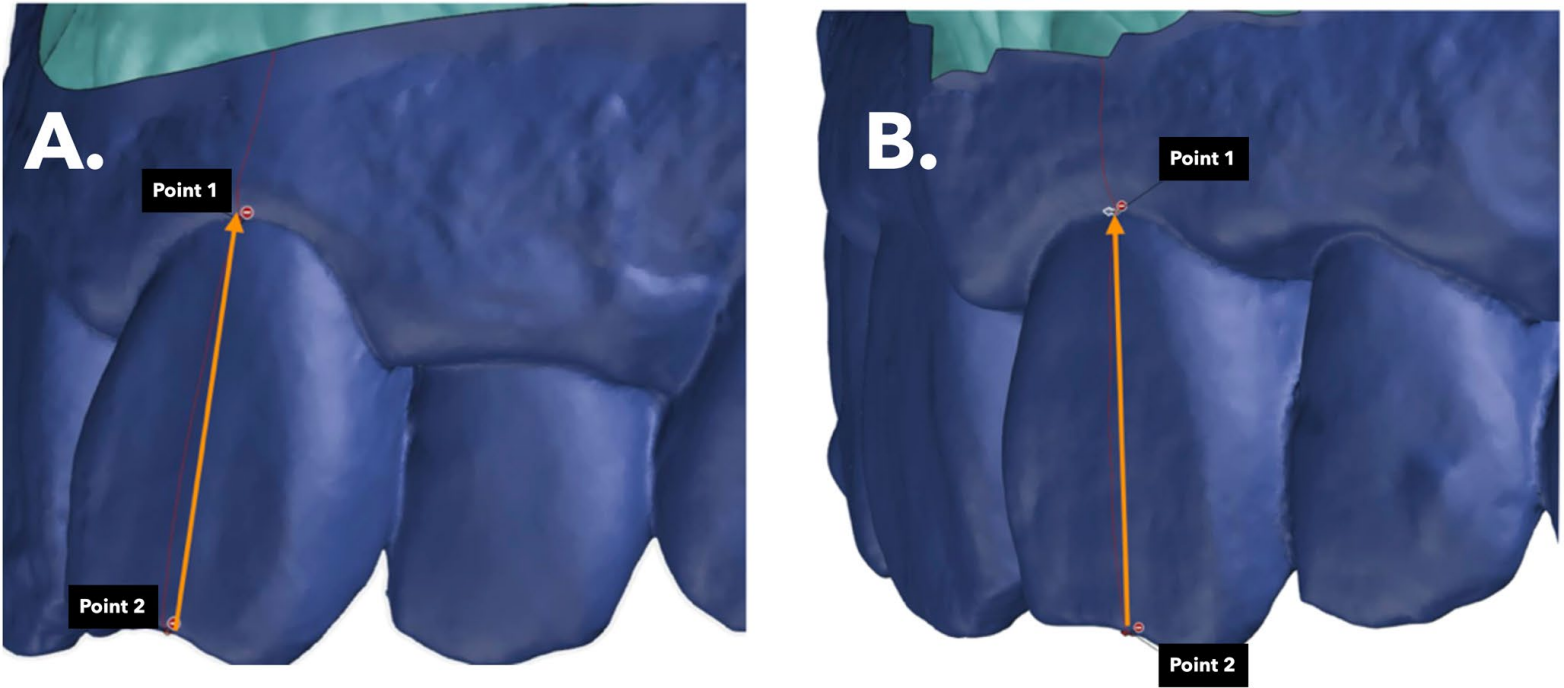
**A** Perpendicular section pre-treatment from the middle of the incisal edge; **B** Perpendicular section post-treatment from the middle of the incisal edge

For dental movement measurement, a sagittal section was created from the midpoint of the incisal edge in both T0 and T1 models. A section was made perpendicular to the axis of the upper incisor, starting from the midpoint of the incisal edge in the pre-treatment model (Fig. 2). After superimposition, movements were quantified: retrusion/protrusion (MOV_SAG), retro/proinclination (INC_SAG), extrusion/intrusion (MOV_VERT), crown inclination (TIP), and rotation (ROT) (Fig. S1 y S2 in Supplementary Information).

### Measurement of gingival changes

To quantify changes in the free gingiva, a reference line was drawn along the axis of the incisor in the pre-treatment model, extending from the midpoint of the incisal edge (P1) to the gingival margin (MG1) at a single point (P1-MG1). A second measurement was taken in the post-treatment model, from the midpoint of the incisal edge (P1) to the gingival margin (MG2) (P2-MG2, T1) (Fig. 2). The difference between these two measurements was calculated, with a negative value indicating apical migration and a positive value indicating coronal migration of the free gingiva. After obtaining data on both tooth movements and gingival changes, statistical analysis was performed to assess correlations and determine whether any specific tooth movement was associated with apical or coronal migration of the free gingiva in the upper incisors of each patient following orthodontic treatment. This method was designed to evaluate relative changes in the position of the free gingival margin between pre- and post-treatment scans, rather than absolute gingival thickness.

### Statistical analyses

A sample size of 124 teeth was required (α = 0.05; power > 0.8) to detect a difference of 0.128 mm, assuming SD = 0.5.

Soft tissue gain/loss (mm) was analyzed using mean, median, SD, minimum, maximum, and range in SPSS v28.0 (IBM Corp., Armonk, NY, USA), considering tooth movements, gender, and age.

Pearson’s correlation coefficient (r) evaluated the relationship between tooth movement and soft tissue variation (*p* < 0.05). Linear regression assessed the effect of incisor proclination/retroclination, with soft tissue change as the dependent variable. Stepwise multivariate regression identified predictors.

Measurements were performed by a single examiner; 10% were verified by a second. Reliability was determined with the intraclass correlation coefficient (ICC).

## Results

A total of 124 upper incisors (teeth 12, 11, 21, and 22) were analyzed to assess the parameters of interest. The sample consisted of 31 patients, including 15 males and 16 females, all of whom underwent orthodontic treatment with clear aligners. The mean age of the participants was 42.9 ± 1.20 years. A total of 62 intraoral scans were collected, 31 before treatment and 31 after its completion. The average orthodontic treatment duration was approximately 18.0 ± 4.5 months.

**Fig. 3:**
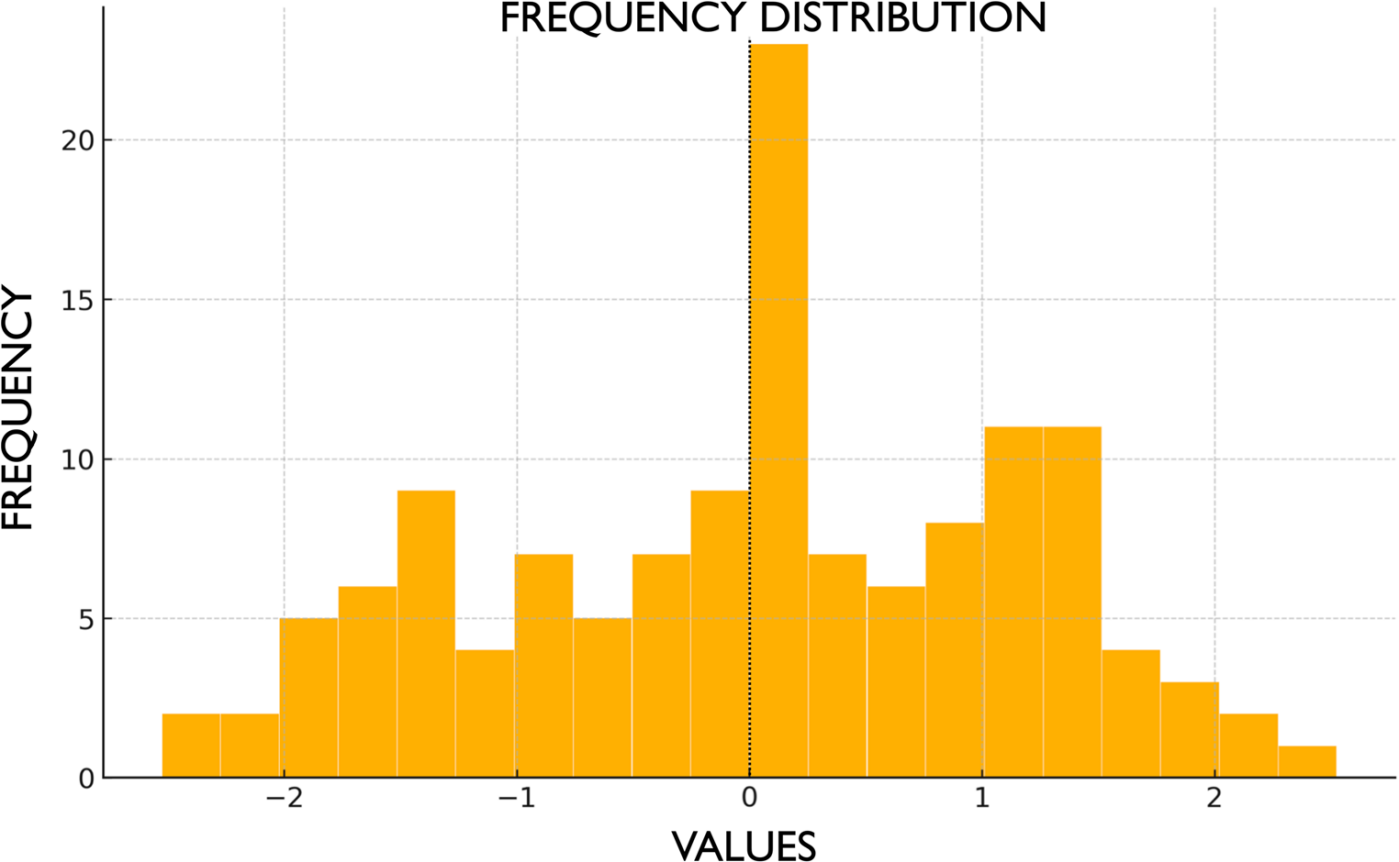
Histogram showing the descriptive distribution of free gingival gain (+) and loss (−)

Interexaminer reliability was assessed using the ICC, with a value exceeding 0.85. To evaluate intraexaminer consistency, the first examiner repeated the measurements on a subset of patients, obtaining an ICC greater than 0.80. High intra- and inter-examiner reliability supports the reproducibility of the measurement protocol. Descriptive analyses of vertical soft tissue gain (+) or loss (−), as well as tooth movement, are presented in Fig. 3; Table 1, respectively.

**Table 1:** Descriptive statistics of dental movements categorized by type and direction

Category	Classification	*N* (%)	Mean	95% CI	Range
MOV_VERT. (mm)	Extrusion	60	3.36	[2.37, 4.34]	[0.19, 5.03]
	Intrusion	64	2.61	[2.21, 3.01]	[0.12, 5.13]
INC_SAG (°)	Proclination	60	14.46	[13.14, 15.78]	[0.33, 23.94]
	Retroclination	64	15.16	[13.55, 16.76]	[3.47, 39.73]
MOV_SAG (mm)	Protrusion	60	3.64	[3.19, 4.08]	[0.44, 7.78]
	Retrusion	63	3.53	[3.15, 3.91]	[0.79, 6.56]
TIP (°)	Mesial crown tipping	66	9.26	[7.54, 10.98]	[0.0, 34.72]
	Distal crown tipping	48	9.35	[6.72, 11.99]	[0.0, 45.01]
ROT (°)	Mesio-palatal rotation	53	19.13	[15.65, 22.61]	[1.43, 66.78]
	Mesio-labial rotation	55	16.99	[14.04, 19.95]	[0.0, 39.34]

Data are presented as number of cases (N), percentage (%), mean values, 95% confidence intervals (CI), and full extent of movement or angulation. Abbreviatures: MOV_VERT., vertical movement — extrusion or intrusion (mm); INC_SAG, Sagittal inclination — proclination or retroclination (°); MOV_SAG, Sagittal translation — protrusion or retrusion (mm); TIP, Crown angulation — mesial or distal tipping (°); ROT, Rotation — mesial-palatal or mesial-vestibular direction (°)

### Relationship between incisor inclination and vertical soft tissue changes (INC_SAG)

The statistical analysis evaluating the association between proclination and vertical soft tissue changes revealed a positive and statistically significant correlation. Proclination movements were associated with marginal gingival gain. The Pearson correlation coefficient was *r* = 0.560 (*p* < 0.05), with the greatest gingival gain observed at approximately ten degrees of proclination (Fig. S3 in Supplementary Information).

Retroclination movements also demonstrated a statistically significant positive correlation, although weaker than that observed for proclination. In this case, retroclination was associated with marginal gingival loss, with a Pearson correlation coefficient of *r* = 0.275 (*p* < 0.05; Fig. S4 in Supplementary Information).

Linear regression analysis indicated that for each degree of proclination, there was an average increase of 0.096 mm in gingival tissue height. Conversely, each degree of retroclination was associated with an average decrease of 0.073 mm in gingival tissue height.

### Relationship between sagittal tooth movements and vertical soft tissue changes (MOV_SAG)

The statistical analysis exploring the association between sagittal movements—specifically protrusion and retrusion—and vertical soft tissue gain or loss did not reveal statistically significant correlations. The Pearson correlation coefficient was *r* = 0.111 for protrusion and *r* = − 0.121 for retrusion (*p* > 0.05 in both cases), indicating that these movements were not significantly associated with gingival tissue height changes.

### Relationship between vertical tooth movements and soft tissue changes (MOV_VER)

The statistical analysis evaluating the association between vertical movements—specifically extrusion and intrusion—and vertical soft tissue gain or loss did not demonstrate statistically significant correlations. The Pearson correlation coefficient was *r* = − 0.014 for extrusion and *r* = 0.021 for intrusion (*p* > 0.05 in both cases), suggesting no meaningful relationship between vertical tooth movement and marginal gingival changes.

### Relationship between crown tipping movements and vertical soft tissue changes (TIP)

The statistical analysis examining the association between crown inclination movements—specifically mesial and distal—and vertical soft tissue gain or loss did not reveal a statistically significant correlation. The Pearson correlation coefficient was *r* = − 0.051 for mesial crown inclination and *r* = 0.052 for distal crown inclination (*p* > 0.05 in both cases), indicating no significant relationship between these tipping movements and marginal gingival changes.

### Relationship between rotational movements and vertical soft tissue changes (ROT)

The statistical analysis evaluating the association between rotational movements—specifically mesial-palatal and mesial-buccal rotations—and vertical soft tissue gain or loss did not reveal a statistically significant correlation. The Pearson correlation coefficient for mesial-palatal rotation movements was *r* = − 0.044, and for mesial-buccal rotation movements, it was *r* = 0.063 (*p* > 0.05 in both cases).

### Association between sex, age, and vertical soft tissue changes

Pearson correlation analysis revealed a moderate positive association between free gingival loss and older age (*r* = 0.216). Linear regression analysis demonstrated that the female sex variable exhibited a negative coefficient in both models (B = -0.935 and B = -0.923, respectively), with high statistical significance (*p* < 0.001). This suggests that, after adjusting for other factors, female sex is associated with greater soft tissue loss, independent of tooth movement.

## Discussion

Research on the impact of orthodontic treatment on soft tissues is crucial. Adjunctive orthodontic therapy often enhances periodontal outcomes by improving tooth alignment [13]. To optimize the benefits of tooth movement and minimize adverse effects, a comprehensive and objective assessment of associated soft tissue changes is essential.

Pre- and post-treatment 3D digital models can be superimposed to detect differences. Misalignment during superimposition may introduce errors in length and volume measurements, increasing proportionally with the degree of misalignment. While overlap errors occur when reference regions are not perfectly identical, such discrepancies are typically minor and do not significantly affect analysis [9].

Palatal rugae may change with growth and development^7^, but this study included patients over 25 years to reduce age-related variability. Following Avella et al. (2010) [10], areas in the third palatal ridge and posterior regions were used, appropriate for patients with extractions.

The study found a positive correlation between age and soft tissue loss, consistent with Mascarenhas et al. (2018) [14], who noted that aging decreases periodontal regenerative capacity and increases susceptibility to inflammation, promoting gingival recession.

Orthodontic movements involving incisor proclination and protrusion were associated with marginal gingival gain, while retroclination and retrusion led to apical migration. These correlations help clarify the relationship between tooth movement and periodontal response.

Although statistically significant associations were observed, the magnitude of the detected soft tissue changes was small. Estimated variations of approximately 0.09 mm per degree of crown inclination may not be directly perceptible in isolation. However, orthodontic treatments typically involve cumulative angular changes over time, and the associated soft tissue response may become clinically relevant when multiple degrees of inclination are considered. Moreover, even minor changes may be relevant in patients with thin periodontal tissues or reduced anatomical tolerance.

These results align with Melsen et al. (2005) [15], who found proclination linked to gingival migration, likely due to buccal alveolar bone thickening. Yared et al. (2006) [16] also reported increased soft tissue stability with proclination. However, these studies lacked quantification, underscoring the value of the present findings.

In contrast, Kobylyanskyy et al. (2024) [1] reported reduced alveolar bone height and marginal gingiva with proclination. Still, they agreed that greater inclination changes caused more pronounced soft tissue alterations, consistent with our observations.

Renkema et al. (2013) [17] documented that retroclination and retrusion cause apical gingival migration, exposing roots and reducing bone thickness. This supports the present study’s findings on the impact of movement direction on marginal gingiva.

Proclination and protrusion may promote gingival gain due to tension in periodontal fibers and bone adaptation. Retroclination and retrusion may compress fibers, lead to bone remodeling, and reduce gingival support, increasing the risk of recession and compromising aesthetics [18].

These findings have clinical relevance. In selected cases, changes in crown proclination and protrusion may be associated with favorable soft tissue trends; however, such movements should be carefully planned and interpreted within the individual periodontal and anatomical context. Retroclination and retrusion should be carefully planned, particularly in cases with limited vestibular bone or thin gingiva. Monitoring for recession is essential, and periodontal-orthodontic combination treatments may help mitigate adverse effects.

Although a weak correlation was found between age and vertical soft tissue gain/loss, other factors likely play a more decisive role. Adult patients were selected to reduce the confounding effect of bone remodeling seen in younger individuals, allowing for a more controlled analysis [19].

This study has certain limitations, including a relatively small sample size and the exclusion of variables such as bone quality, and initial root position, which are known to influence alveolar bone dimensions [20]. Inclination measurements were derived exclusively from crown morphology using STL files. Consequently, this approach does not allow direct assessment of root position, root axis, or the relationship between the root and the alveolar cortical plates. Since periodontal outcomes such as dehiscence and gingival recession are influenced by root displacement relative to the cortical plates, the present findings should be interpreted as associations between changes in crown inclination and free gingival position rather than as direct evidence of root movement.

It should be noted that the proposed measurements reflect relative changes in the clinically visible position of the gingival margin derived from STL-based models and do not allow isolation of specific biological changes of the free (marginal) gingiva. Despite this limitation, changes in crown inclination represent the clinically visible component of orthodontic movement and may serve as a useful surrogate marker for identifying patients at increased risk of soft tissue alterations, particularly in situations where three-dimensional imaging is not justified.

Other confounding factors were not directly evaluated in the present study, including gingival phenotype. Although the influence of gingival phenotype on periodontal outcomes is well recognized, recent consensus reports have highlighted the subjectivity and heterogeneity of its clinical assessment, which relies on multiple parameters measured using different techniques and thresholds [21–23]. Consequently, patients were not stratified according to gingival phenotype in order to avoid classification bias. Therefore, the present findings should be interpreted as indicative of systematic trends in soft tissue response rather than as evidence of clinically perceptible changes at the individual tooth level.

Nonetheless, the study has strengths. It is the first to evaluate not only inclination but also mesiodistal inclination, rotation, intrusion, and extrusion, beyond prior research focused mainly on inclination [1, 24, 25]. A novel method using STL model superimposition is introduced to quantify soft tissue height changes without requiring CBCT [26, 27]. The study emphasizes an interdisciplinary periodontal-orthodontic approach and performs individualized three-dimensional assessments of each maxillary incisor, surpassing previous two-dimensional studies that lacked incisor specificity [28].

## Conclusions

- A novel digital method was developed to quantify dental movements based on crown morphology and relative changes in free gingival height using STL model superimposition.
- Changes in crown retroclination, older age, and female sex were associated with apical migration of the free gingiva.
- Changes in crown proclination were associated with coronal migration of the free gingiva.
- These findings describe correlational relationships between orthodontic tooth movement patterns and periodontal soft tissue changes and should be interpreted with caution, as STL-based measurements do not allow direct assessment of root position, root displacement, or alveolar bone morphology.

## Supplementary Information

Below is the link to the electronic supplementary material.

Supplementary Material 1

## Data Availability

The datasets used and/or analyzed during the current study are available from the corresponding author upon reasonable request.
