# Supplementary material for: Soft tissue changes in upper incisors from tooth movements using a new measurement method based on digital scans superimposition in adult patients
Source: Clin Oral Investig. 2026 Apr 23;30(5):191. doi: 10.1007/s00784-026-06873-0 (PMC13106264; doi:10.1007/s00784-026-06873-0)

Soft tissue changes in upper incisors from tooth movements using a new measurement method based on digital scans superimposition in adult patients.

Luis Sastre-Buades; Verónica García-Sanz; Beatriz Tarazona-Álvarez; Natalia Zamora-Martínez; Sara Camañes-Gonzalvo; José María Montiel-Company; Vanessa Paredes-Gallardo; Carlos Bellot-Arcís.

Online Resource

Clinical Oral Investigations

Correspondence to: Verónica García-Sanz, Orthodontics Teaching Unit, Department of Stomatology (University of Valencia), Gascó Oliag, 1. 46010. Valencia, Spain.

e-mail: veronica.garcia-sanz@uv.es

Fig. S1 A. Pre-treatment sagittal section (2.1); B. Post-treatment sagittal section (2.1). C. Superposition of the pre- and post-treatment sagittal slices of the 2.1

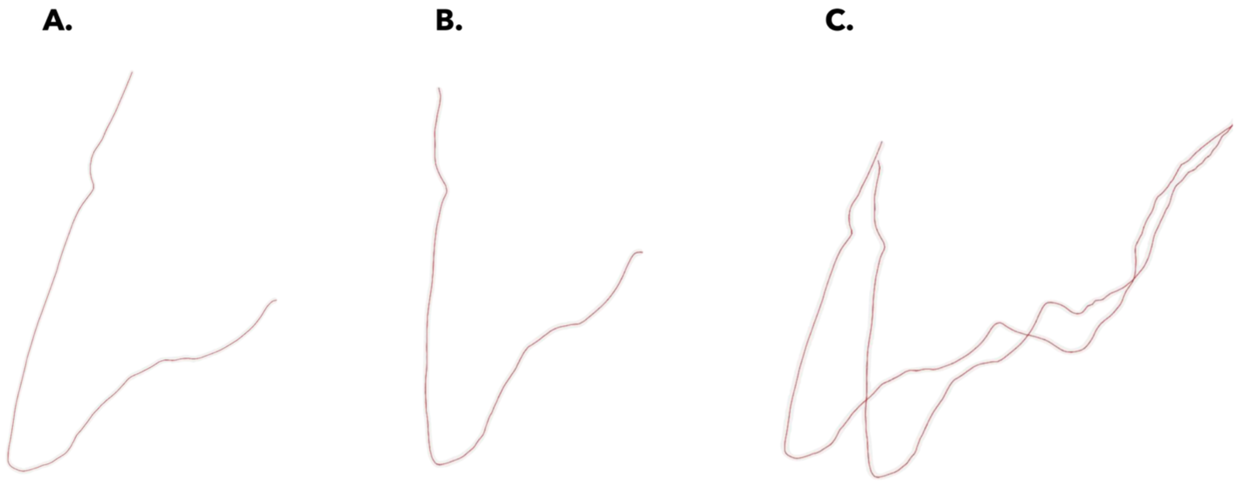

Fig. S2 A. Protrusive/retrusive movement measurement (mm); B. Retroclination/proclination measurement (°); C. Tip movement measurement (°); D. Rotation movement measurement (°); E. Intrusion/extrusion movement measurement (mm).

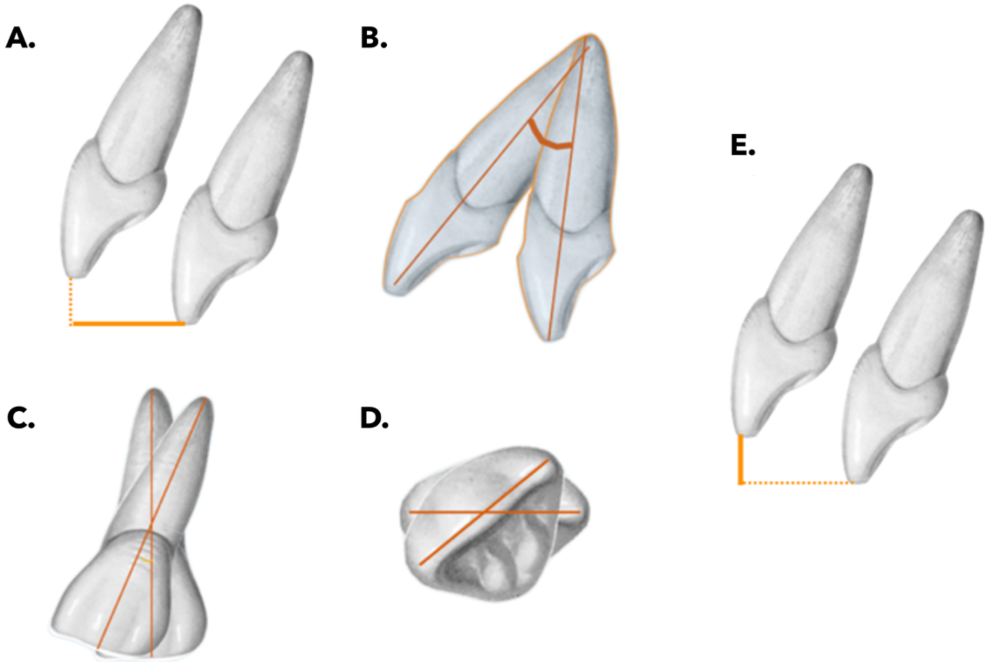

Fig. S3 Correlation between free gingival gain/loss (+/-) and proclination movement.

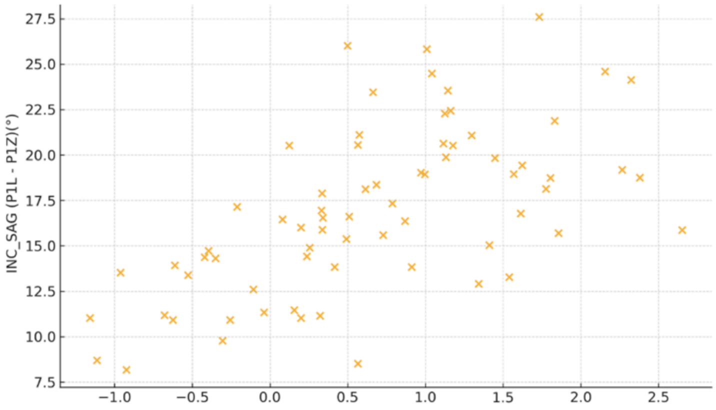

Figure S4. Correlation between free gingival gain/loss (+/-) and retroclination movement.

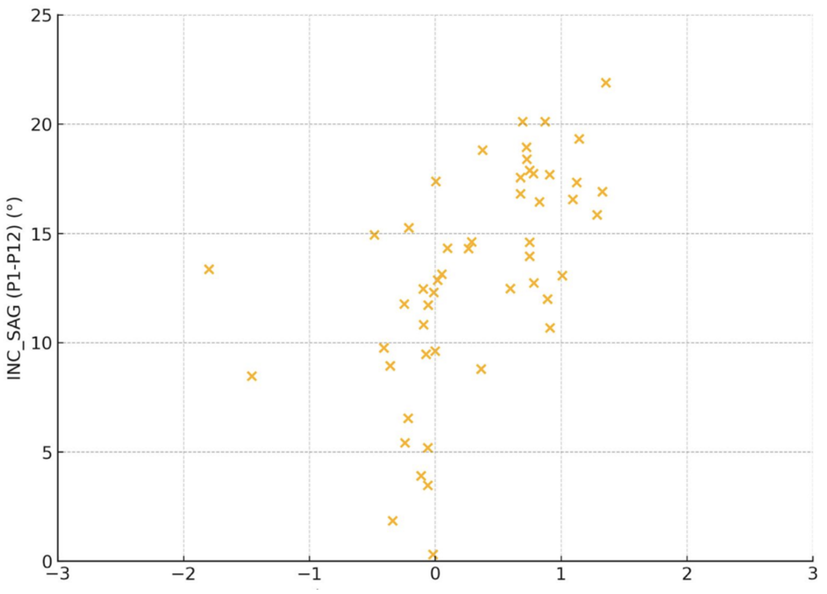

Supplement: Supplementary file 1 — Supplementary Material 1 [file 784_2026_6873_MOESM1_ESM.pdf]
